# Endovascular procedures cause transient endothelial injury but do not disrupt mature neointima in Drug Eluting Stents

**DOI:** 10.1038/s41598-020-58938-z

**Published:** 2020-02-07

**Authors:** Anouchska Autar, Aladdin Taha, Richard van Duin, Ilona Krabbendam-Peters, Dirk J. Duncker, Felix Zijlstra, Heleen M. M. van Beusekom

**Affiliations:** 1 Department of Cardiology, Erasmus MC, University Medical Center Rotterdam, Rotterdam, The Netherlands; 2 Department of Neurology, Erasmus MC, University Medical Center Rotterdam, Rotterdam, The Netherlands; 3 Department of Hematology, Erasmus MC, University Medical Center Rotterdam, Rotterdam, The Netherlands

**Keywords:** Cardiovascular biology, Interventional cardiology

## Abstract

Extensive application of coronary intravascular procedures has led to the increased need of understanding the injury inflicted to the coronary arterial wall. We aimed to investigate acute and prolonged coronary endothelial injury as a result of guidewire use, repeated intravascular imaging and stenting. These interventions were performed in swine (N = 37) and injury was assessed per coronary segment (n = 81) using an Evans Blue dye-exclusion-test. Scanning electron microscopy and light microscopy were then used to visualize the extent and nature of acute (<4 hours) and prolonged (5 days) endothelial injury. Guidewire and imaging injury was mainly associated with denudation and returned to control levels at 5 days. IVUS and OCT combined (Evans Blue staining 28 ± 16%) did not lead to more acute injury than IVUS alone (33 ± 15%). Stent placement caused most injury (85 ± 4%) and despite early stent re-endothelialization at 5 days, the endothelium proved highly permeable (97 ± 4% at 5 days; p < 0.001 vs acute). Imaging of in-stent neointima at 28 days after stent placement did not lead to neointimal rupture. Guidewire, IVUS and OCT induce acute endothelial cell damage, which does not increase during repeated imaging, and heals within 5 days. Interestingly, endothelial permeability increases 5 days post stenting despite near complete re-endothelialization.

## Introduction

Intravascular imaging using Intravascular ultrasound (IVUS) and optical coherence tomography (OCT) yields detailed information of vascular architecture and may enhance efficacy of percutaneous coronary interventions (PCI)^1^. IVUS and OCT are considered safe^2–4^, but imaging can induce endothelial injury and, while rare, can induce thrombotic complications^5^. This could be of importance with respect to antithrombotic protection when employing repeated imaging strategies. In a similar fashion, interventions such as stent placement, mechanical thrombectomy and other arterial probing techniques are expected to yield injury^6^.

Manipulation such as stents and guiding catheters are known to induce vascular injury^7,8^. Guiding catheters can induce sustained caliber changes in the radial artery up to one year after its use as access for coronary angiography^8^. Bare metal stents (BMS) show endothelial dysfunction for at least three months following stenting in healthy coronary arteries^7^ and this is possibly worse in drug eluting stents (DES)^9^.

While imaging catheters are small in diameter and more flexible than guiding catheters, it is unknown to what extent they induce endothelial injury, whether they differ from each other, whether repeated imaging increases this injury, and how injury heals over time. Moreover, neointima in stents and especially in DES can be thin and potentially pro-thrombogenic^5^.

We therefore set out to assess acute injury induced by different intravascular imaging modalities in comparison to guidewire placement and direct stenting. The extent of vascular injury and subsequent healing was assessed acutely and, in a smaller set, at 5 days follow-up by dye-exclusion testing and scanning electron microscopy (SEM). In addition, to test intimal fragility, we assessed acute imaging-induced injury of in-stent neointima at 28 days following DES and BMS placement, using light microscopy.

## Materials and Methods

### Animals and preparation

Experiments were performed in farm-bred swine (Yorkshire-Landrace, 30–35 kg, N = 37). The study was approved by the animal ethics committee of the Erasmus MC University Medical Center Rotterdam and complied with the “Guide for the care and use of laboratory animals” (NIH publication 85–23) and reported according to ARRIVE guidelines^10^. Animals were pretreated with dual antiplatelet therapy (DAPT; 300 mg Acetyl Salicylic Acid (ASA; Aspegic, Sanofi, Paris, France) and a loading dose of 300 mg Clopidogrel (Plavix, Sanofi Aventis, Gouda, The Netherlands)) one day prior to the procedure. Animals were sedated by Ketamine (20 mg/kg, Alfasan, Woerden, The Netherlands) and midazolam (3 mg/kg, Deltaselect, Dreieich, Germany). Anesthesia was induced by Thiopental (10–15 mg/kg; Pentothal, Hospira Enterprises BV, Hoofddorp, The Netherlands) and maintained by Isoflurane (1–2.5% volume; Nicholas Piramal Limited, London, England) as guided by heart rate and blood pressure. Antibiotic prophylaxis was administered by an intramuscular injection of Streptomycin-Penicillin Procaine (0.1 mg/kg; Streptoprocpen 20/20, Eurovet, Bladel, The Netherlands). Animals that were allowed to recover from anesthesia, were returned to the animal care facilities for postoperative recovery. During follow-up, DAPT (300 mg ASA and 75 mg Clopidogrel) was administered daily in accordance with clinical practice for stent placement. ASA also served as analgesic.

### Materials for interventions

The following materials were used for the interventions: Guidewire (Pilot 50, Abbott Vascular, Santa Clara, CA, USA), IVUS catheter (Atlantis SR pro, 40 MHz, Boston Scientific, Natick, MA, USA) and OCT catheters (C7 Dragonfly^TM^ St Jude Medical, St Paul, MN, USA aided by a Twin-pass catheter, Vascular Solutions Inc. Minneapolis MN, USA).

The following stents were used: Xience V (Everolimus eluting stent, Abbott Vascular, Santa Clara, CA, USA), Biomatrix (Biolimus eluting stent, Biosensors Int. Singapore), Focus NP (Sirolimus eluting stent, Envision Ltd. Surat, India) and Amazonia Croco (BMS, Minvasys, Gennevilliers, France).

### Experimental design

**Figure 1:**
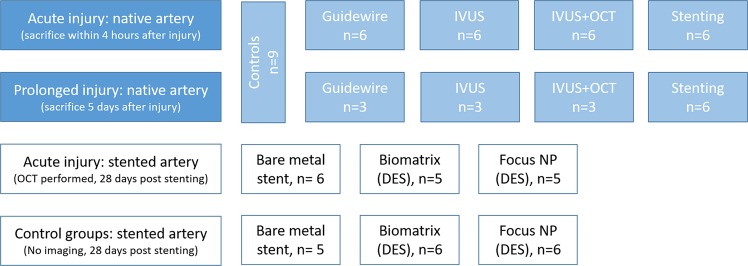
Flow chart study groups. The flow chart displays the number of studied segments per intervention for the acute injury group in native arteries and the prolonged injury group in native arteries. It also displays the stented segments used to study acute neointimal injury caused by OCT-imaging 28 days post stenting and the control segments 28 days post stenting in which no OCT-imaging was performed. IVUS = Intravascular ultrasound; OCT = optical coherence tomography. DES = Drug eluting stent.

Study groups are summarized in Fig. 1. We defined four groups of injury by type of intervention: (1) guidewire only, (2) IVUS, (3) IVUS followed by OCT, and (4) stent-induced injury, which we studied in native arteries. Injury was studied acutely, and at 5 days following intervention to assess persistence of injury. One type of injury was induced per coronary artery using a random block design as described before^11^ to allow even distribution of the interventions between the arteries. As a control for pre-existing endothelial permeability, a total of 9 segments were left untreated, taken from the acute and prolonged injury group. In addition, we studied acute imaging induced injury (OCT) of the neointima in 16 stented arteries at 28 days following DES or BMS placement (6 Amazonia Croco, 5 Biomatrix and 5 Focus NP). As a control group, another 17 stented arteries were studied 28 days post placement without undergoing imaging (5 Amazonia Croco, 6 Biomatrix and 6 Focus NP).

### Interventional procedures

Following a carotid arterial cut-down, an introducer sheath was placed in the common carotid artery for arterial access and 10.000 IU Heparin and 250 mg ASA were administered intra-arterially. Following administration of Isosorbide-di-nitrate (Cedocard, Takeda, Hoofddorp, The Netherlands), arterial segments were selected in each of the coronary arteries in preparation for the interventional procedures, under guidance of quantitative angiography (CAAS II, PIE Medical, Maastricht, the Netherlands). The used arteries are LAD (proximal and middle branch, segment 6, 7), LCX (left circumflex branch, segment 11 and 13) and RCA (proximal, middle and distal branch, segments 1, 2 and 3). The IVUS catheters were positioned according to standard clinical practice, the guidewire withdrawn in the guiding catheter and the IVUS catheter left in place for 90 seconds of dwell time, to mimic the duration of a regular pull-back. For sequential imaging the guidewire was re-advanced, the IVUS catheter exchanged for the OCT catheter and advanced to the same location for repeated imaging. In the guidewire only group, the guidewire was similarly positioned and left in place for about 90 sec. To exclude unwanted late injury by guiding catheters, angiography at follow-up was not performed. In the control segments, no wires or catheters were introduced. Stents were placed with a stent:artery ratio of 1.1:1(and a duration of balloon inflation of 45 seconds. After completion of imaging or stenting, the final quantitative angiogram was performed and in follow-up animals, the guidewire and the introducer sheath were removed, their carotid artery ligated and the skin closed in two layers.

### Dye exclusion test and tissue harvesting

**Figure 2:**
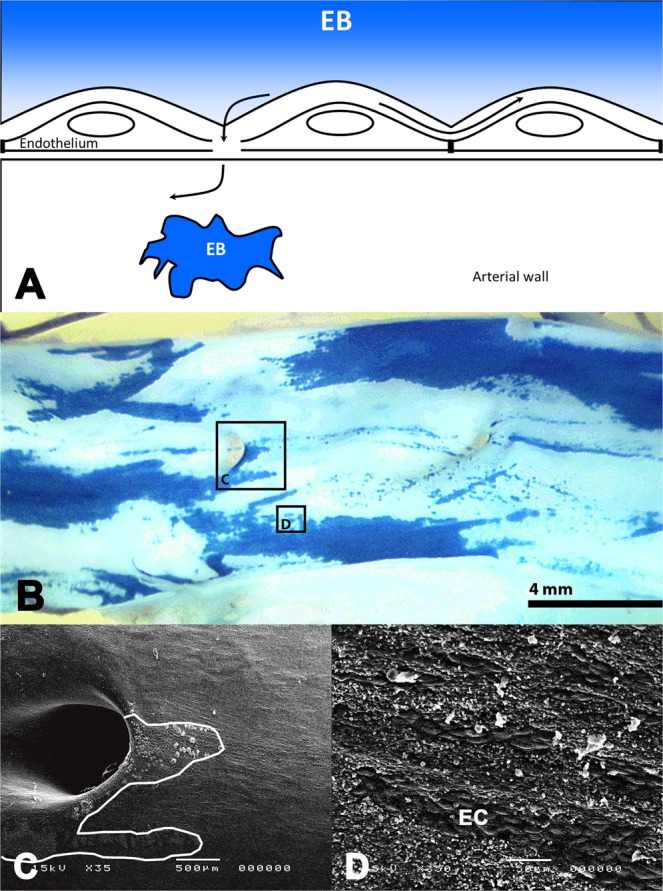
Evans Blue Vascular Damage. Challenging the vasculature with Evans Blue (EB) following vascular imaging reveals areas of endothelial cell permeability or denudation by staining the arterial wall blue. Underneath a functional endothelium, the arterial wall remains unstained (**A**,**B**). Scanning electron microscopy (**C,D**) shows that permeability is mainly caused by overt denudation (i.e. endothelial cell loss), resulting in penetration of the blue dye. The line delineates the area stained blue in B. D shows islands of intact endothelial cells (EC).

At the designated follow-up time the depth of anesthesia was increased and the thorax opened by a midsternal split. Then the arteries were subjected to a dye-exclusion test as described before^7,12^. In short, 150 to 200 ml 0.3% (w/v) Evans Blue (EB, Sigma-Aldrich) in saline was infused directly into the coronary circulation following aortic clamping and a saline flush to remove blood. The dye will penetrate only the areas where the endothelium is permeable (Fig. 2), allowing passage of the small EB and staining the injured surface blue. Where the endothelium is intact, an unstained surface will be maintained. After completion of EB-saline-infusion, the coronary arteries were flushed with 300 ml saline to remove the dye before pressure fixation *in situ* (approximately 100 mmHg) with 500 ml electron microscopy fixative (4% buffered paraformaldehyde and 2% glutaraldehyde). Then the heart was excised and the treated and control segments were dissected from the epicardial surface.

### Morphometry

The excised segments were opened longitudinally, assessed under a dissection microscope for penetration of the blue dye and documented in digital format. Using computer assisted planimetry (Clemex vision PE, Clemex Technologies Inc. Longueuil, Canada), endothelial injury in the imaged segments was quantified by determining both blue (injured) and white (uninjured) areas. Injury was expressed as the percentage blue stained area within the imaged segment. All arteries were subsequently processed and analyzed by SEM using routine techniques^7^ except for the arteries with mature NI that were analyzed by routine histology (see below). Analysis of endothelial morphology in stained and unstained areas by SEM was guided by macroscopy of the blue dye and using side branches as geometric landmarks (Fig. 2).

### Histology

Imaging of mature NI and the differential response to imaging of DES and BMS was studied by light microscopy to assess vascular damage and the subsequent inflammatory response. Presence of micro thrombi, leukocyte adhesion, platelets and oedema were scored proximal, mid and distally within the stent. The proximal and distal section in the stent were taken approximately 500 µm from the stent edge.

### Statistical analysis

All data are given as mean ± SD or median (min-max). Morphometric data for Evans Blue permeability was analyzed with SPSS (SPSS Inc. version 25). Intergroup differences were assessed using one-way ANOVA followed by post-hoc analysis with a Bonferroni correction in case of statistical significance. For injury assessed by light microscopy, we determined the number of positive segments per stent for each of the measured parameters, which was used as the unit of analysis. Semi quantitative data were analysed using the Kruskal-Wallis test and Mann-Whitney U test.

## Results

### Acute endothelial injury by Evans Blue

**Figure 3:**
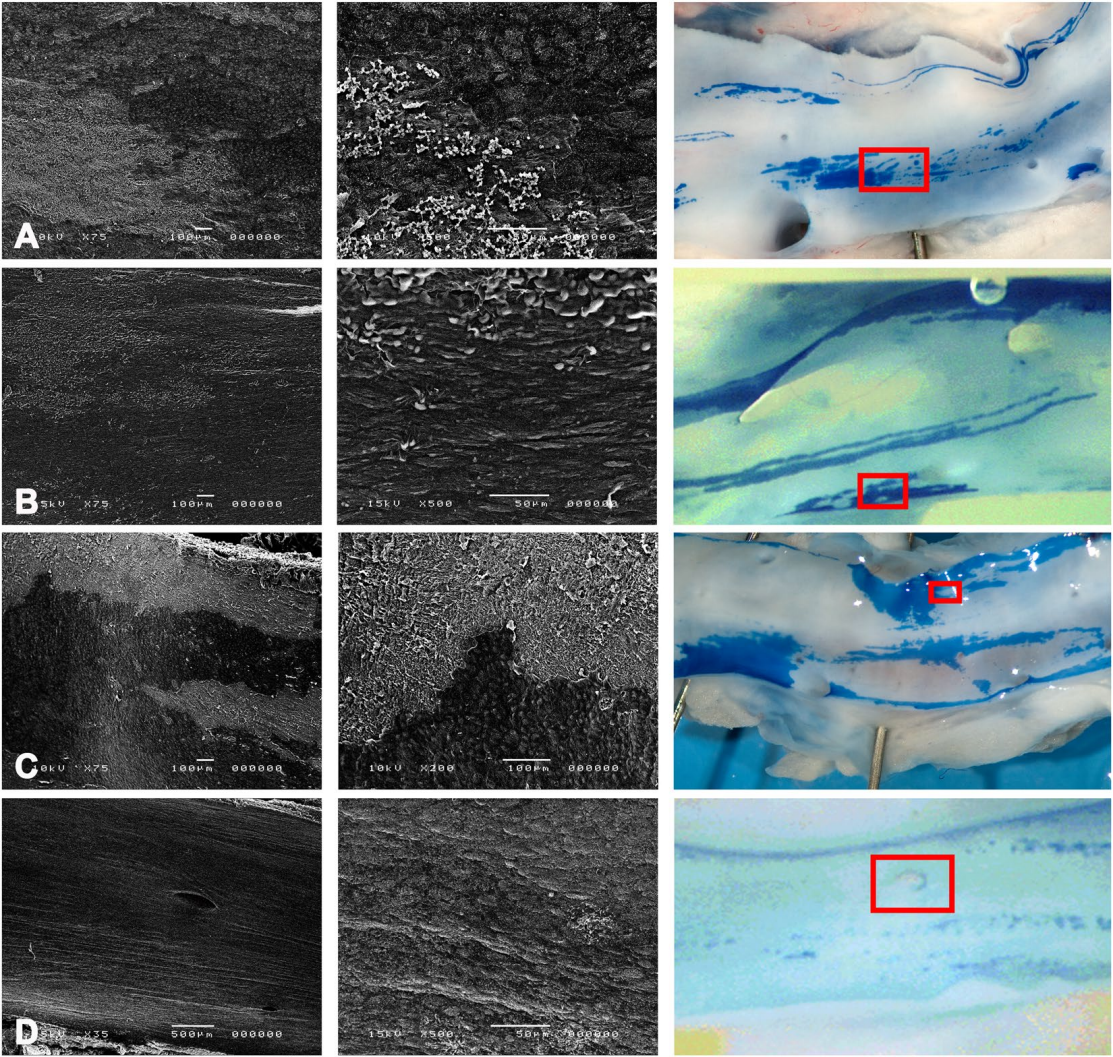
Vessel wall injury. Scanning electron microscopy overviews, details in relation to the Evans Blue dye-exclusion test (right) showing vascular injury by means of different imaging catheters. Images show that vascular imaging as well as engaging an artery with a guidewire induce vascular injury. (**A**) Guidewire; (**B**) IVUS imaging; (**C**) IVUS + OCT imaging, (**D**) control artery without injury. IVUS = Intravascular ultrasound; OCT = optical coherence tomography.

**Figure 4:**
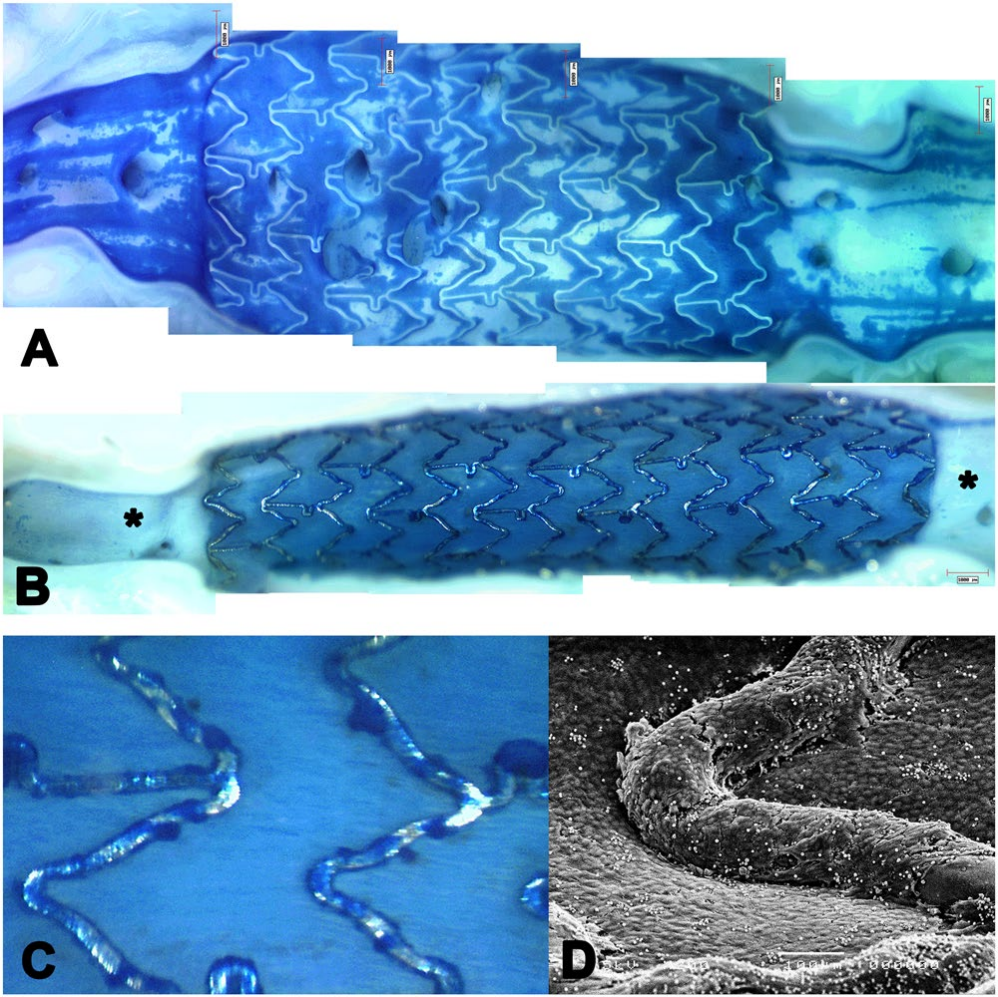
Vessel wall injury after stenting. Evans Blue Dye-exclusion acute (**A**) and at 5 days following stent implantation (**B**). The non-stented adjacent segments clearly indicate areas of damage inflicted by the guidewire and the stent delivery system during stent placement. At 5 days, the injury inflicted by the guidewire and delivery system has been repaired (*) while stent induced permeability is still present both between and over the stent struts, despite a near complete endothelial stent strut coverage (**C,D**).

All animals completed the study and data is given in Table 1 and illustrated in Figs. 3 and 4. Vessels treated with a catheter or guidewire showed more acute injury than the control group (p < 0.01). Sequential imaging after guidewire usage by IVUS and OCT did not result in an increase in the amount of staining as compared to IVUS alone. As expected, stenting induced significantly more injury (85%, p < 0.001 vs. all), but islands of undamaged endothelium were still clearly present (Fig. 4A).

**Table 1:** Percentage vessel wall injury per intervention group by Evans Blue.

Group	Acute Injury	n	Prolonged injury (5d)	n
Guidewire	18.15 [7.70–38.70]*	6	0.70 [0.10–4.70]^†^	3
IVUS	32.94 [17.74–43.90]*	6	1.10 [0.80–2.10]^†^	3
IVUS + OCT	28.00 [13.70–43.80]*	6	2.30 [1.80–2.50]^†^	3
Stent respectively S/A ratio	84.67 [81.01–90.33]** respectively 1.10 ± 0.02	6	99.41 [91.37–100.00]^†^ respectively 1.09 ± 0.05	6
Control	0.17 [0.00–1.30]			9

Morphometric analysis of injury as determined by the percentage Evans Blue staining of the area exposed to imaging, data given as median[min-max]. S/A ratio is given as mean ± SD. Immediately after the procedure all catheters induce injury and after 5 days the remaining injury caused by guidewire or imaging catheter use is minimal, unlike permeability after stenting. *p < 0.01 vs. control; **p < 0.001 vs. all acute injury; ^†^p < 0.05 vs. acute injury. IVUS = Intravascular ultrasound; OCT = optical coherence tomography, S/A ratio = stent/artery ratio.

### Prolonged endothelial injury

Data is summarized in Table 1 and illustrated in Fig. 4. At 5 days follow-up, EB staining of all imaged segments had reduced to background levels except for sites of extensive guiding catheter induced damage of the ostium. The differences between imaging catheters and the control were not present anymore. Injury after stenting however was still significantly different (p < 0.001 vs. all). Actually, stenting showed an increase in permeability as compared to acute injury (p < 0.001), which was mainly due to an increased permeability between the stent struts (Fig. 4B,C).

### SEM of acute and prolonged injury

In general, SEM showed that EB staining was associated with either overt endothelial denudation or injured but present endothelium, whereas unstained areas showed an intact endothelium. Examples for each of the catheters are given in Fig. 3 and show that guidewire injury was restricted to the endothelium and did not induce deep injury. Similarly, the imaging catheters did not inflict severe injury to the arterial wall, although they did induce platelet adhesion. The prolonged injury, visible at 5 days following imaging and stenting, is illustrated in Fig. 4 and consisted mostly of activated endothelium (leukocyte adhesion, surface folds) as overt denudation was no longer observed. Even in the stents, re-endothelialisation was near complete overlying the struts. We did observe (once) more deep injury at the tip of a Twin-pass used for wire exchange.

### Imaging of mature NI

**Figure 5:**
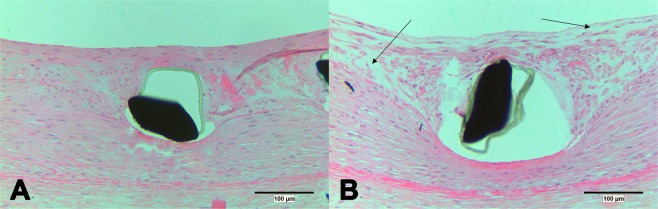
Histology after stenting. Microscopy of control (**A**) and imaged (**B**) stent segment showing acute oedema (arrows) as a result of imaging induced denudation. Haematoxylin Eosin stain.

**Figure 6:**
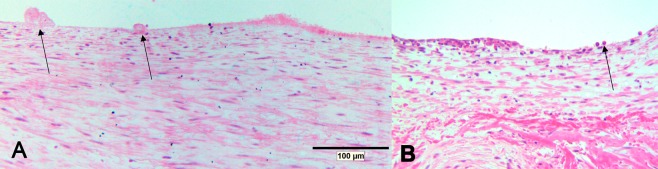
Denudated segments. Microscopy of denuded segments showing microthrombi (**A**, arrow) and leukocyte adhesion (**B**, arrow). Haematoxylin Eosin stain.

Despite the fact that the NI was extremely thin in some DES, imaging did not disrupt this thin NI, often classified as fragile. Overall, imaging resulted in removal of endothelium and subsequent localized oedema formation (Fig. 5). Oedema formation was observed both in BMS and DES alike. In addition, we observed the formation of micro thrombi and adhesion of leukocytes (Fig. 6). In non-imaged control stents, denudation of endothelium was not observed and formation of platelet carpets, leukocyte adhesion and oedema were observed significantly less (p = 0,002; p = 1,7E-9; p = 1,2E-8, respectively). There were no statistical differences between the three different stents. Semi quantitative scoring of these parameters is given in Table 2.

**Table 2:** Semi quantitative assessment of in-stent neointimal injury by light microscopy.

Parameter	BIO + OCT	BIO − OCT	Focus + OCT	Focus − OCT	BMS + OCT	BMS − OCT
S/A ratio	1.12 ± 0.03	1.10 ± 0.03	1.08 ± 0.04	1.08 ± 0.04	1.13 ± 0.02	1.06 ± 0.05
Microthrombi	2/15	1/18	1/15	0/18	1/15	0/15
Leukocyte adhesion	7/15	2/18	4/15	1/18	4/15	0/15
Platelet carpets	13/15	0/18	15/15	0/18	15/15	0/15
Oedema	11/15	2/18	12/15	0/18	12/15	0/15

The following unwanted sequelae of neointimal injury were scored in each proximal, middle or distal stent segment: presence of microthrombi, leukocyte adhesion, platelet carpets and oedema. S/A ratio is given as mean ± SD. Other data given as number of positive segments per total segments analyzed. Statistical analysis shows that imaging leads to a significant increase of leukocyte adhesion, platelet carpets and oedema, as compared to non-imaged controls. There was no difference found between the three different stents. BIO = Biomatrix, Focus = Focus NP, BMS = Bare metal stent, OCT = Optical Coherence Tomography, S/A ratio = stent/artery ratio.

## Discussion

The use of intracoronary imaging or pressure measurements to guide PCI is considered good clinical practice^13^. Indeed, coronary imaging yields detailed information of the vascular architecture, and coronary pressure measurements were shown to improve clinical results^14^. In this preclinical study, we investigated the vessel wall injury caused by frequently used imaging modalities and stenting during PCI on a microscopic level.

While clinicians are aware that manipulation results in vascular injury, to date, it is still unknown to what extent intravascular imaging, and especially sequential imaging, induces coronary injury and how this injury heals over time. IVUS imaging alone induced endothelial injury of approximately 30% of the surface area. Importantly, we found that sequential imaging did not increase the area of endothelial injury. Injury caused by intravascular imaging and guidewires healed relatively fast and the increased permeability returned to baseline values within 5 days.

Stent implantation is more traumatic for the arterial vessel wall. Acute injury is significantly higher and persists longer than for the other catheters examined. The damage is not restricted to the inner layer of the vessel wall but is likely also deeper. Five days after implantation, when re-endothelialisation in these models is well underway towards completion, it even showed a significant increase in permeability despite endothelial coverage. This might for instance be due to increased stiffness of the artery^15^, the presence of thrombus and inflammatory cells, and the chronic injury inflicted on the vasculature by stents^16,17^.

Imaging of DES could be considered a risky undertaking since DES have long been associated with delayed healing, thin NI coverage and increased thrombogenicity^18^. Surprisingly, imaging of DES did not disrupt the NI, despite the fact that it was extremely thin in some DES. Classification of a thin NI as fragile should therefore perhaps be reconsidered. In clinical practice BMS are still preferred over DES, in specific patient groups, for example when surgical interventions are planned and early discontinuation of DAPT is needed. However, it has been argued that risk of late stent thrombosis in DES is nowadays so low that DES should be preferred over BMS in all cases^19,20^. Our data support this point-of-view.

Regardless, it might be considered good clinical practice to pre-treat elective patients with DAPT and keep them on this regimen for a while, to prevent thrombotic complications following imaging of all stents and scaffolds. The current study indicates that less than 5 days are needed for vascular healing following imaging in swine coronary arteries. Assuming that diseased arteries heal slower, antithrombotic prophylaxis during a period of approximately two-to-three weeks might be considered a safe period following intravascular imaging^21^.

Endothelial denudation is an integral part of many animal models of atherosclerosis and mural thrombosis can be involved in plaque growth. As such, denudation might be considered to pose a risk in increasing restenosis. While repeated guiding catheter changes using the radial approach have been associated with incremental structural and functional changes to the vasculature^22^, intravascular imaging induces less damaging injury. Data from heart transplant patients who are subjected to repeated IVUS imaging have shown immediate and 1 year safety^23,24^ and, similarly, OCT has shown a good safety profile^2,4^. Indeed, our data show that the injury that is inflicted during vascular imaging and guidewire manipulation is mostly superficial and heals within a short time frame, while guiding catheter injury remains clearly visible. In addition, imaging of in-stent NI did not show a response that exceeded injury of normal arteries. While this response may be exaggerated in an atherosclerotic model, we do not expect imaging to lead to adverse events. Indeed, given the data from the literature, it is not expected that this superficial and temporary endothelial denudation will result in more clinical restenosis in PCI treated segments.

## Conclusion

All of the tested vascular imaging modalities as well as guidewire manipulation induce acute endothelial cell damage, which does not increase during repeated imaging and heals within 5 days. Interestingly, endothelial permeability increases 5 days post stenting despite near complete re-endothelialisation in DES.

Imaging of mature in-stent neointima in DES does not induce neointimal disruption.
